# Estimation of Topical Glaucoma Medication Over-Prescription and Its Associated Factors

**DOI:** 10.3390/jcm13010184

**Published:** 2023-12-28

**Authors:** Eri Takao, Aona Ichitani, Masaki Tanito

**Affiliations:** Department of Ophthalmology, Shimane University Faculty of Medicine, Izumo 693-8501, Japan

**Keywords:** glaucoma medication, medication adherence, over-prescription, older age, hyperopia, polypharmacy, unit-dose medication

## Abstract

This study aims to report the disparity between the ideal and actual quantities of eyedrops prescribed to individual glaucoma patients. This retrospective observational study included 676 patients receiving treatment with antiglaucoma topical medication(s) in at least one eye. These patients had follow-up appointments scheduled at mean intervals of 3.4 ± 1.4 months and were actively using antiglaucoma medication. The mean age was 70.4 ± 11.9 years, with 372 (55%) being male. The over-prescription volume was 1.4 ± 1.7 bottles per month for each medication when prescribed for both eyes. Multiple regression analysis revealed that older age (*p* = 0.03), hyperopic refractive error (*p* < 0.0001), and the use of multiple medications (*p* = 0.03) were associated with a larger over-prescription volume, while the use of unit-dose medication only (*p* < 0.0001) was associated with a smaller over-prescription volume. Factors such as sex, Mini-Cog cognitive function score, best-corrected visual acuity, intraocular pressure, glaucoma type, and a history of cataract surgery were not significantly associated. This study revealed a significant over-prescription of eyedrops for glaucoma patients, with actual prescriptions often exceeding the theoretically ideal amount by 2.4 times, influenced by factors like age and the format of prescriptions, where unit-dose eyedrops show promise in reducing excess.

## 1. Introduction

Glaucoma is a group of ophthalmic neurodegenerative diseases in which the optic nerve is damaged, resulting in visual field constriction and vision loss [1]. Glaucoma affects 76 million people worldwide and is expected to increase to 95 million by 2030 due to an aging population and increased diagnostic opportunities [2]. Elevated intraocular pressure (IOP) is a major risk factor for glaucoma. The only evidence-proven treatment for glaucoma is to lower IOP through medication, laser, or incisional surgery [3,4], although the medication applied topically is the mainstay of the treatment option in the majority of glaucoma patients [5]. Oral medications are frequently used in fields other than ophthalmology. They are dispensed in single-use formulations, making it easier for both patients and physicians to manage the number of medications. In contrast, by convention, glaucoma topical medications are prescribed in bottles with the amount of drug used over a certain period of time, resulting in variations in drug usage among patients. The skillful use of glaucoma eyedrops varies among patients and is not easy to predict [6,7,8].

The cost of glaucoma medication therapy is the highest among all ophthalmic medication therapies [9,10]. In the USA, through Medicare Part D in 2013, the total cost attributed to glaucoma medications accounted for 54% of the total medication cost and 72% of the total volume [9]. In a retrospective, longitudinal cohort study conducted from 2015 through 2016 in the USA, glaucoma medications comprised 42.7% of all ophthalmic medication expenditures, followed by dry eye medications at 29.5% [10]. In Japan, among the 100 most frequently prescribed ophthalmology medications in 2019, 32 were glaucoma medications [3]. In England, by analyzing prescribing cost data held by the NHS Business Authority from 2000 to 2012, the number of glaucoma medication prescriptions dispensed increased by 67% in 2012 compared to 2000, with medication costs increasing by 88% over the same period [11]. The cost of glaucoma treatment is a significant component of visual impairment-related costs, with medication costs contributing substantially [12]. Therefore, understanding the actual amount of glaucoma drugs prescribed and controlling prescriptions, especially if they are being over-prescribed, is a crucial concern from the perspective of healthcare economics. In addition, prescription requirements may be influenced by insurance plans, income, and co-payments in individual cases, which in turn may affect treatment adherence.

In the literature, there are limited data available regarding the extent to which drug prescriptions exceed the ‘ideal amount’ on an individual basis. This study aims to examine the actual quantity of drugs prescribed for individual glaucoma cases and to elucidate the variance from the standard prescribed quantity. Furthermore, we conducted statistical analyses to identify factors associated with the disparities between these quantities.

## 2. Subjects and Methods

### 2.1. Study Design and Subjects

This retrospective study was conducted in accordance with the principles outlined in the Declaration of Helsinki and the Ethical Guidelines for Medical and Health Research Involving Human Subjects in Japan. The Institutional Review Board (IRB) of Shimane University Hospital meticulously reviewed and granted approval for this research (Approval No. 20220616-1, issued on 21 July 2022). The IRB approval did not necessitate written informed consent from each patient for publication. Instead, the study protocol was made available at the study institutions, allowing participants to opt out if they wished to do so. Subjects were recruited consecutively at the Department of Ophthalmology, Shimane University Hospital, spanning from April 2020 to March 2022. All subjects who met the inclusion criteria were enrolled in the study. These criteria encompassed patients receiving treatment with antiglaucoma topical medication(s) in at least one eye, patients having scheduled follow-up appointments with intervals of at least 2 weeks, and patients with recorded cognitive function scores determined using the Mini-Cog assessment.

### 2.2. Measurements

The following data were collected through a comprehensive review of the subjects’ medical charts: each subject’s age, sex, cognitive function, best-corrected visual acuity (BCVA) obtained using a decimal chart, spherical equivalent refractive error (SERE), intraocular pressure (IOP), glaucoma type, history of cataract surgery, number of glaucoma medication bottles used, use of unit-dose (single-use) medications, duration until the next visit, and volume of prescribed medication. Cognitive function was estimated using the Mini-Cog test, with a scale ranging from 0 (poor) to 5 (good); a score of 2 or worse was considered indicative of possible cognitive impairment [13]. Our institution routinely administers cognitive function tests to glaucoma clinic patients. The decimal BCVA was converted into the logarithm of the minimum angle of resolution (LogMAR). Respective counting fingers, hand motions, light perception, and no light perception values were considered as the decimal visual acuities of 0.0025, 0.002, 0.0016, and 0.0013 [14]. SERE was measured via autorefractometry (TonoRef III, Nidek, Gamagori, Japan), and IOP was measured via Goldmann applanation tonometry.

### 2.3. Calculation of Over-Prescription Volume

The actual prescription volume per month was determined by dividing the prescribed volume (in bottles) by the duration until the next visit (in months). The prescribed volume was the value recorded in the medical record. The standard prescription volume was defined as 1 bottle per month for both eyes, which equates to 0.5 bottle for a single eye. For unit-dose medications, 30 units were considered equivalent to 1 bottle for once-per-day regimen medications (e.g., Tapros Mini Ophthalmic Solution, Santen Osaka, Japan, and Eybelis Mini Ophthalmic Solution, Santen). Similarly, 60 units were considered equivalent to 1 bottle for twice-per-day regimen medications (e.g., Cosopt Mini Ophthalmic Solution, Santen). The over-prescription volume was calculated by subtracting the standard prescription volume from the actual prescription volume. A positive value for the over-prescription indicated overprescribing. To standardize the value for both eyes, the over-prescription volume of medications prescribed for a single eye was multiplied by 2. For patients using multiple medications, the calculation was performed for each medication, and the mean value was calculated for each patient. Consequently, the obtained over-prescription volume was standardized among subjects per month, per both eyes, and per each medication.

### 2.4. Statistical Analysis

The data were presented as mean ± standard deviation (SD) with 95% confidence interval (CI) ranges for continuous parameters, and in numbers and percentages for categorical parameters. For continuous parameters, the potential association with the over-prescription volume was evaluated through linear regression analysis with Pearson’s correlation coefficient. For categorical parameters, groups were compared concerning the over-prescription volume using unpaired *t*-tests. Additionally, potential associations with the over-prescription volume and various parameters, including age, sex, Mini-Cog score, BCVA, SERE, IOP, glaucoma type, cataract surgery, number of medication use, and exclusive use of unit medication, were explored through multiple regression analysis. All statistical analyses were conducted using JMP Pro statistical software version 16.1.0 (SAS Institute, Inc., Cary, NC, USA). A *p*-value of less than 0.05 was considered statistically significant.

## 3. Results

Demographic data for the subjects are presented in Table 1. The mean age of all 676 subjects was 70.4 ± 11.9 years, with 372 (55%) being male and 304 (45%) being female. Cognitive function, as assessed via the Mini-Cog test, indicated that 53 subjects (7.8%) had cognitive function impairment, defined by a Mini-Cog score of ≤2. Half of the subjects had primary open-angle glaucoma (PG, 52.6%), while approximately one-fourth had exfoliation glaucoma (EG, 23.8%) and other types of glaucoma (23.6%). Half of the subjects were treated with a single anti-glaucoma medication (49.3%), while the rest used multiple medications (50.7%). A total of 69 subjects (10.2%) were treated exclusively with unit-dose medication(s), while the majority (89.8%) were treated solely with regular bottled medication(s) or a combination of unit-dose and bottled medication(s). In all subjects, during the mean visit interval of 3.4 months, the over-prescription volume (i.e., the difference between standard prescription volume and actual prescribed volume) was calculated to be 1.4 ± 1.7 bottles per month for each medication when prescribed for both eyes. This suggests that the actual prescribed volume was 2.4 times greater than the standard prescription volume.

**Figure 1:**
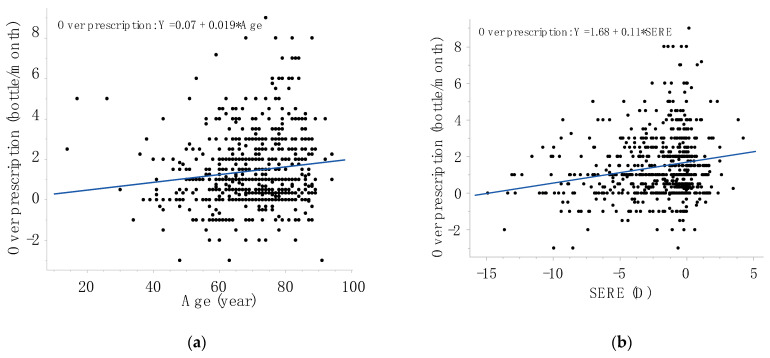
Effects of age (**a**) or refractive error (**b**) on over-prescription. Regression equations are obtained via linear regression analysis. SERE, spherical equivalent refractive error.

The associations between over-prescription volume and various parameters, as calculated using univariate analysis, are presented in Table 2 and Table 3. Among continuous parameters, older age (*p* = 0.0007) and hyperopic SERE (*p* < 0.0001) were associated with a larger over-prescription volume, while BCVA and IOP were not significantly associated (Table 2). Associations between age (Figure 1a) or SERE (Figure 1b) and over-prescription volume are illustrated in scatter plots, where most subjects required a greater volume of medication than the standard prescription volume (i.e., plotted above the y-axis zero line). Among categorical parameters, a history of cataract surgery (*p* = 0.02) and the use of multiple medications rather than monotherapy (*p* = 0.006) were associated with a larger over-prescription volume, while using unit-dose medication exclusively as opposed to other medication regimens (*p* < 0.0001) was associated with a smaller over-prescription volume (Table 3). Differences in sex, Mini-Cog score, and glaucoma type were not significantly associated with the over-prescription volume (Table 3).

**Figure 2:**
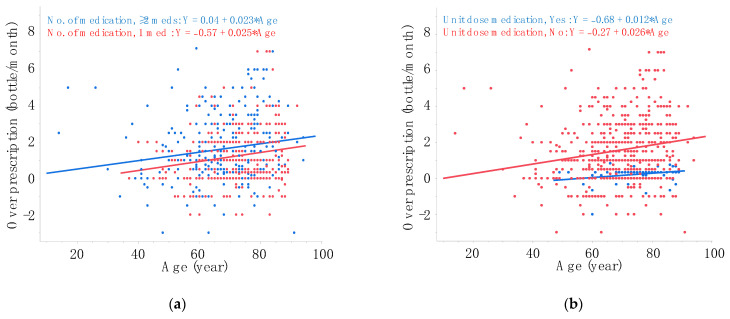
Effects of age on over-prescription in groups stratified by number of medication (**a**) or use of unit-dose medication (**b**). Regression equations are obtained via linear regression analysis. SERE, spherical equivalent refractive error.

A multiple regression analysis was conducted to further explore the possible associations between over-prescription volume and the parameters (Table 4). The analysis indicated that, once again, older age (*p* = 0.03), hyperopic SERE (*p* < 0.0001), the use of multiple medications (*p* = 0.03) was associated with a larger over-prescription volume, while using unit-dose medication exclusively (*p* < 0.0001) was associated with a smaller over-prescription volume. Notably, a history of cataract surgery was not associated with over-prescription volume in the multivariate analysis (*p* = 0.5), suggesting that the significant association observed in univariate analysis was a result of age-related covariate effects (i.e., older subjects were more likely to have a history of cataract surgery than younger subjects). Scatter plots (Figure 2a) demonstrate the association between age and over-prescription volume in groups stratified by the number of medication used, showing that over-prescription volume increased with age in both groups. Similarly, scatter plots (Figure 2b) illustrate that the effect of age on over-prescription volume was evident in subjects using bottled medications but was virtually absent in subjects using unit-dose medication exclusively.

## 4. Discussion

In this study, we calculated the over-prescription amount (difference between the standard prescription amount and the actual prescription amount) when prescribed for both eyes to be 1.4 ± 1.7 per month for each medication, with the actual prescription amount being 2.4 times the standard or ideal prescription amount. It was also found that older age, less myopic SERE, and multiple drug use resulted in larger over-prescription amounts, while the use of only unit-dose medications resulted in smaller over-prescription amounts.

While patient backgrounds, survey methodologies, and definitions of failure varied, it has been observed that between 10% and 90% of patients struggle with the correct application of eyedrops [15]. Prior studies [7,15,16,17,18,19,20,21,22] have identified key factors contributing to this challenge, including the need for multiple applications, inadvertent contact between the bottle tip and eye or surrounding areas, and difficulties in targeting the conjunctival sac. Notably, patients who inaccurately placed eyedrops outside the eye tended to require more prescription bottles monthly compared to those adept at eyedrop application [23]. Furthermore, the practice of applying multiple drops in a single session not only led to wastage, but also increased healthcare expenses [24]. A notable trend observed was the higher mean age in the group failing to use eyedrops correctly, especially in a sitting position, compared to those who succeeded [25]. Factors previously identified as increasing the risk of failure in eyedrop application include older age, female gender, coexisting arthritis, significant visual impairment, lower visual acuity even after correction, reduced self-efficacy, limited educational background, and the absence of proper training in eyedrop administration [15,18,26]. Consequently, as patients age, their declining physiological and motor abilities reasonably heighten the need for more prescribed medications [18].

In our previous study, 56% of glaucoma patients failed eyedrop instillation by subjective assessment with video recordings [7]. In that study, older age, a lower cognitive function score, less myopic objective refractive error, and a lower visual field foveal threshold were the factors in failures [7]. In earlier studies, factors such as poorer corrected VA and inferior VF defects were linked to difficulties in administering eyedrops [26]. This was primarily due to patients’ challenges in visualizing the tip of the medication bottle. When using topical eyedrops, it is necessary to remove glasses, which may make it difficult for individuals with hyperopia to see the tip of the eyedrop bottle. The difficulty in seeing the tip of the eyedrop bottle may explain the reason for the association between hyperopic SRER and over-prescription. A device to improve visibility, such as changing the color of the tip of the eyedrop bottle, may be an effective measure to reduce over-prescription.

The use of multiple medication was associated with a larger over-prescription volume in this study. In our previous video-assessed study, 17% of glaucomatous eyes even failed to instill eyedrops into the conjunctival sac [7]. The failure to properly instill medication into the conjunctival sac might pose a greater risk in the treatment of glaucoma compared to other causes of instillation failure [17,22]. This issue often results in not achieving the targeted IOP, necessitating the prescription of additional medications. Usually, the second and third medications were prescribed because of insufficient efficacy of the initial medication. In such patients, there is a high likelihood that some of them were prescribed multiple medications because of the inability of the first medication use. Under these circumstances, even without sufficient reduction in IOP, patients may face increased burdens, including higher medical costs and a greater risk of adverse events associated with the use of additional medications. To avoid unnecessary over-prescription, it is required to establish a system that verifies the appropriateness of eyedrop instillation techniques before adding additional medications. This study found that the use of unit-dose medication is an effective way to reduce the over-prescription of glaucoma medications. This strategy seemed even more effective in older patients. Each unit-dose medication is sufficient in terms of volume, as one unit can contain at least six drops. As with oral medications, unit-dose eyedrops can be easily counted and may be useful in maintaining adherence. Unit-dose medication is generally more expensive than bottled medication but not twice as expensive. It is important to note that unit-dose medication has other problems such as more plastic waste and a difficulty to squeeze the bottles for some patients. The number of commercially available unit-dose ophthalmic solutions is still few, with only three ophthalmic solutions available in Japan. The development of unit-dose eyedrops should be promoted for the sake of adherence and the medical economy.

In the United States, data from the American Academy of Ophthalmology’s Intelligent Research in Sight Registry revealed a significant rise in the number of annual minimally invasive glaucoma surgery (MIGS) procedures during the study period, increasing from 7586 in 2013 to 39,677 in 2018 [27]. Concurrently, there was a modest decline in the number of standard glaucoma surgeries, from 16,215 in 2013 to 13,701 in 2018 [27]. In Japan, in nationwide trends in glaucoma surgical procedures assessed by using the NDB Open Data, incisional and laser glaucoma surgeries increased 180% and 111%, respectively, from 2014 to 2020 [4]. In Germany, by analyzing the quality report of hospitals, the number of glaucoma procedures performed increased by 75% from 27,811 in 2006 to 48,794 in 2018 [28]. Recent research has underscored the safety and effectiveness of SLT as an initial treatment option for glaucoma [29,30]. The European Glaucoma Society now recognizes SLT as either a primary or supplementary treatment for open-angle glaucoma and ocular hypertension, highlighting its clinical importance [31,32]. In Australia, while glaucoma medication prescriptions reached their highest in 2015, there was a subsequent decrease of 14.9% by 2017 [33]. This decrease coincided with a marked increase in glaucoma laser therapies, drainage device implantations, and trabecular microbypass surgeries during the same timeframe [33]. Therefore, in patients with risk factors for over-prescription, IOP-lowering therapy that does not depend on adherence, such as MIGS or SLT, may be a promising treatment option not only for IOP reduction, but also for economic aspects.

This study had several limitations. Like other retrospective studies, this study should include patient selection bias. The study cite was a tertiary care center to which patients were referred for surgery after inadequate IOP reduction with eyedrops. In addition, this study was conducted in an area of Japan with a particularly aged population. For these reasons, this study might have overestimated the amount of over-prescription compared to the general glaucoma population. In this study, we set the shortest visit intervals at two weeks. Given that the minimum prescription unit is one bottle, cases with visit intervals of less than one month may potentially overestimate over-prescription. However, as there were only two cases with visit intervals of less than one month, we believe that the overall impact on the study results is minimal. In Japan, under the public insurance, the burden rate for prescribed medications was lower in children and older patients. Thus, at least in part, a lower out-of-pocket cost might explain the larger over-prescription in older patients. Therefore, the present results may differ in regions with different insurance systems.

## 5. Conclusions

This study revealed that glaucoma patients were prescribed an excess of 1.4 bottles (2.4 times) of eyedrops per month compared to the theoretical prescription amount. In addition to patient factors such as older age and hyperopia, the prescription form of eyedrops is a factor affecting the difference in prescription volume. Single-drug therapy using fixed-dose combination or single-use formulations may be useful in reducing the excessive number of eyedrops prescribed.

## Figures and Tables

**Table 1:** Demographic data.

Parameters	N or Mean ± SD	% or 95% CI Range
Subjects		
Age, years	70.4 ± 11.9	69.5, 71.3
Sex		
Male	372	55.0
Female	304	45.0
Mini-Cog score		
≥3	623	92.2
≤2	53	7.8
BCVA, LogMAR	0.2 ± 0.4	0.2, 0.3
SERE, D	−2.1 ± 3	−2.3, −1.8
IOP, mmHg	16.7 ± 5.5	16.3, 17.1
Glaucoma type		
PG	352	52.6
EG	159	23.8
Other	158	23.6
Cataract surgery		
No	285	42.2
Yes	391	57.8
Number of medication use, bottle		
≤1	333	49.3
≥2	343	50.7
Unit-dose medication only		
No	607	89.8
Yes	69	10.2
Visit intervals, months	3.4 ± 1.4	3.3, 3.5
Over-prescription volume (bottle/month)	1.4 ± 1.7	1.3, 1.6

SD, standard deviation; CI, confidence interval; BCVA, best-corrected visual acuity; LogMAR, logarithm of minimal angle of resolution; SERE, spherical equivalent refractive error; D, diopter; IOP, intraocular pressure; PG, primary open angle glaucoma; EG, exfoliation glaucoma. Over-prescription was standardized per month, per both eyes, and per each medication.

**Table 2:** Possible associations between over-prescription volume and various continuous parameters calculated via univariate analyses.

Parameter	Estimate	95% CI	*p* Value
Age, year	0.02 ± 0.01	0.06, 0.2	0.0007 *
BCVA, LogMAR	0.2 ± 0.2	−0.04, 0.1	0.3
SERE, D	0.1 ± 0.02	0.1, 0.3	<0.0001 *
IOP, mmHg	−0.02 ± 0.01	−0.1, 0.02	0.1

The correlation is calculated with Pearson’s correlation coefficient. * *p* < 0.05. CI, confidence interval; BCVA, best-corrected visual acuity; LogMAR, logarithm of minimal angle of resolution; SERE, spherical equivalent refractive error; D, diopter; IOP, intraocular pressure.

**Table 3:** Possible associations between over-prescription volume and various categorical parameters calculated via univariate analyses.

Parameter	Mean ± SD	Mean ± SD	*p* Value
Sex	Male, 1.5 ± 0.1	Female, 1.4 ± 0.1	0.5
Mini-Cog score	≥3, 1.4 ± 0.7	≤2, 1.6 ± 0.2	0.5
Glaucoma type	PG, 1.4 ± 0.09	EG, 1.7 ± 0.1	0.2
	Other, 1.4 ± 0.1		
Cataract surgery	No, 1.3 ± 0.1	Yes, 1.6 ± 0.09	0.02 *
Number of medication use, bottle	≤1, 1.3 ± 0.1	≥2, 1.6 ± 0.09	0.006 *
Unit-dose medication only	No, 1.6 ± 0.07	Yes, 0.2 ± 0.2	<0.0001 *

*p* values are calculated using the unpaired *t*-test. * *p* < 0.05. SD, standard deviation; PG, primary open angle glaucoma; EG, exfoliation glaucoma.

**Table 4:** Possible associations among over-prescription volume and various parameters analyzed using a multiple regression model.

Parameter	Estimate	95% CI	*p* Value
Age, year	0.02	0.002, 0.03	0.03 *
Sex, female/male	−0.01	−0.1, 0.1	0.9
Mini-Cog score, ≤2/≥3	0.1	−0.4, 0.6	0.6
BCVA, LogMAR	0.2	−0.2, 0.5	0.3
SERE, D	0.1	0.05, 0.2	<0.0001 *
IOP, mmHg	−0.01	−0.04, 0.01	0.3
Glaucoma type			
PG/EG + other	0.02	−0.2, 0.2	0.8
EG/PG + other	0.1	−0.1,0.3	0.3
Cataract surgery, yes/no	−0.04	−0.2, 0.1	0.5
Number of medication use, ≥2 bottle/1 bottle	0.1	0.02, 0.3	0.03 *
Unit-dose medication only, yes/no	−0.7	−1,−0.5	<0.0001 *

*p* values are calculated using a multiple regression model. * *p* < 0.05. BCVA, best-corrected visual acuity; LogMAR, logarithm of minimal angle of resolution; SERE, spherical equivalent refractive error; D, diopter; IOP, intraocular pressure; PG, primary open angle glaucoma; EG, exfoliation glaucoma.

## Data Availability

Data are fully available upon reasonable request to the corresponding author.
